# Recent Advances in Electronic Skins with Multiple-Stimuli-Responsive and Self-Healing Abilities

**DOI:** 10.3390/ma15051661

**Published:** 2022-02-23

**Authors:** Quanquan Guo, Xiaoyan Qiu, Xinxing Zhang

**Affiliations:** State Key Laboratory of Polymer Materials Engineering, Polymer Research Institute, Sichuan University, Chengdu 610065, China; quanq.guo@gmail.com (Q.G.); qxy@stu.scu.edu.cn (X.Q.)

**Keywords:** electronic skins, self-healing, multimodal sensing, dynamic crosslinking

## Abstract

Wearable electronic skin (e-skin) has provided a revolutionized way to intelligently sense environmental stimuli, which shows prospective applications in health monitoring, artificial intelligence and prosthetics fields. Drawn inspiration from biological skins, developing e-skin with multiple stimuli perception and self-healing abilities not only enrich their bionic multifunctionality, but also greatly improve their sensory performance and functional stability. In this review, we highlight recent important developments in the material structure design strategy to imitate the fascinating functionalities of biological skins, including molecular synthesis, physical structure design, and special biomimicry engineering. Moreover, their specific structure-property relationships, multifunctional application, and existing challenges are also critically analyzed with representative examples. Furthermore, a summary and perspective on future directions and challenges of biomimetic electronic skins regarding function construction will be briefly discussed. We believe that this review will provide valuable guidance for readers to fabricate superior e-skin materials or devices with skin-like multifunctionalities and disparate characteristics.

## 1. Introduction

In recent decades, wearable electronic skins (e-skin), which could transform environmental stimulus variation into electronic signal change, have gained increasing attentions owing to their promising applications in healthcare monitoring, soft robotics, human-machine interfaces, artificial intelligence and protheses fields [1,2,3,4,5,6]. As an indispensable part of Internet of Things (IoTs), e-skin realize seamless integration and harvest useful information between individuals and their surroundings, greatly facilitating the process of intelligent family, community, and country. Until now, various stimulus-responsive E-skins have been extensively developed, such as force, temperature, humidity, analytes, metabolites and electrolytes [7,8,9,10]. However, the single-stimulus detection cannot meet the increasing demands of flexible E-skins for different application scenarios. To mimic the stimulus-perceptive characteristics of human skin, innovative sensing materials and fabrication technology have been studied to explore multi-stimuli-responsive sensors, which could simultaneously monitor diverse stimuli from the external environment and human body [11,12,13,14,15,16]. Despite brilliant achievements, there are still challenges that need to be overcome for their practical applications. For example, the cross-coupling effect from different response signals becomes the main stumbling block for these multifunctional sensor systems. Additionally, the limited sensing performances, lacking of high-performance sensing materials and shortage of rational designing strategies also restrict their further developing evolution.

On the other hand, transformative advances in multiple-stimuli-responsive E-skins should be not just from innovating efficient cross-coupling or better processing techniques for novel sensing materials, but also developing new functionality that improve both working stability and cyclic utilization. Electronic sensors inevitably suffer from mechanical fractures or scratches under repeated bending or stretching, which could cause signal instability and even entire device breakdown. Fortunately, the autonomous recovery features of human skin enlighten researchers to endow e-skin with self-healing ability to restore their original functionalities and mechanical properties after mechanical damage [17,18,19,20,21,22]. However, in most self-healing sensing materials, it is still an enormous challenge to solve the contradiction between mechanical performance and healing efficiency [23,24,25,26,27,28,29,30]. The incomplete recovery of the e-skin would give rise to the changed electric signal response and thereby generate distorted feedback from users. Therefore, efficient, fast, and autonomous self-healing materials and corresponding versatile construction strategies are highly desirable for high-performance self-recovering e-skin and devices.

In this article, we will review recent advances in the multi-stimuli-responsive e-skin with self-healing abilities, focusing on the material design strategies to imitate these important features of biological skins. In the first section, we summarize the successful progress in multifunctional electronic sensors with the capacity to simultaneously detect multiple stimuli and their main challenges are critically analyzed. Subsequently, the second section highlights the effective fabrication approaches for self-healing wearable electronics based on material chemistry and micro/nano-structure regulation strategies. Finally, we conclude the review with a future perspective and development trend of multifunctional biomimetic e-skin.

## 2. Multiple-Stimuli-Responsive E-Skin

As the basic protective and sensitive interface between the external world and body’s sensorium system, the human skins have the unparalleled ability to perceive environmental stimulus change. Taking advantages of skin’s specialized composition and structure including diverse stimulus-receptors, epidermal-dermal ridge structures, afferent nerves, hair and fingerprint, the input multiple stimuli could be effectively amplified and accurately discriminated by the creatures [10]. For the purpose of imitating this sensory ability of human skin, different kinds of types of electronic sensors with the capacity to detect external stimulus have been massively fabricated, including resistance-, capacity-, piezoelectric-, triboelectric-, and potentiometric-type sensors [31,32,33,34,35]. Normally, these stimuli could be mainly divided to physical forces (such as strain, pressure, shearing, bending, torque, and vibration), physical chemistry (such as temperature, humidity, pH) and biochemistry parameters (such as sodium, chlorine, potassium, glucose and lactate) [9]. However, most previously reported e-skin materials were confined to the single-stimulus responsiveness and show limited perceptibility to multiple external factors variation. The exploration of multiple-stimuli-responsive e-skin materials could not only enrich the material species diversity, but also enhance the competitive advantages over other smart devices, even the biological skins. Normally, there are two working principles for the multiple stimuli responsive e-skins. The first one is integrating different single-stimulus-responsive sensors in one sensing platform. Multiple stimuli signals could be directly collected by means of recording the electric signal variations of different sensors. The second one is adopting special stimulus-responsive material that could generate electric signal change in response to multiple stimuli variation. Taking advantages of signal decoupling technology, it has access to gain information about multiple environmental factor changes. Although tremendous advances have been achieved, there is still a grand challenge derived from the cross-coupling effect among the disparate answer signals in response to multiple stimuli. In this part, the representative material design strategies and signal decoupling technology are analyzed in detail, mainly including multidirectional force, multiple physical chemistry stimuli, and biochemical signals sensing materials.

### 2.1. Multidirectional Force Sensing Materials

Force sensors, which can covert external force stimuli into electrical signals, have gain considerable attentions in recent years owing to their prospective utilization in skin-like electronic devices for healthcare monitoring, biomedical protheses, humanoid robot and human-machine interaction applications [36]. According to the difference in measured force directions, flexible force sensors could be mainly classified into strain and pressure sensors. Strain sensors principally measure external forces in planar or longitudinal direction, while pressure sensors function in perpendicular and transverse direction. Based on different working principles, diverse flexible force-responsive sensing materials have been reported via introducing functional nanomaterials in the supporting polymer substrate with specific micro/nano-structure design [2,37,38]. Generally, these nanomaterials cover low-dimensional carbons (e.g., carbon blacks (CBs), carbon nanotubes (CNTs), graphene, MXenes) [39], metal-based conductive fillers (e.g., silver nanowires (Ag NW), gold nanoparticles, liquid metal) [40], conducting polymer-based fillers (e.g., polyaniline (PANI), polypyrrole (PPY), polythiophene, poly(3,4-ethylenedioxythiophene): polystyrene sulfonate (PEDOT:PSS)) [41] and their hybrid conductive fillers (CNTs/graphene hybrid filler) [42]. As for flexible and stretchable supporting polymer matrix, elastic rubbers, fibers, fabrics, and foams are the popularly used ones for flexible force-sensitive sensors [5,9,11]. Up to date, enormous efforts have been devoted to improving the important sensing parameters of wearable force sensors via different chemical and morphological structure design, such as sensitivity, stretchability, hysteresis and response time [43,44,45,46,47]. Nevertheless, if state-of-the-art flexible e-skin are highly desirable, other skin-like force-response performance should be taken into consideration. For example, human skin is highly sensitive to detect external force, allowing simultaneous perception and discrimination of multidirectional tactile stimuli, such as stretching, pressure, bending, texture, normal and shear force. However, it is still a tough task to accurately detect and differentiate multidirectional force stimuli in a single sensing material system.

**Figure 1:**
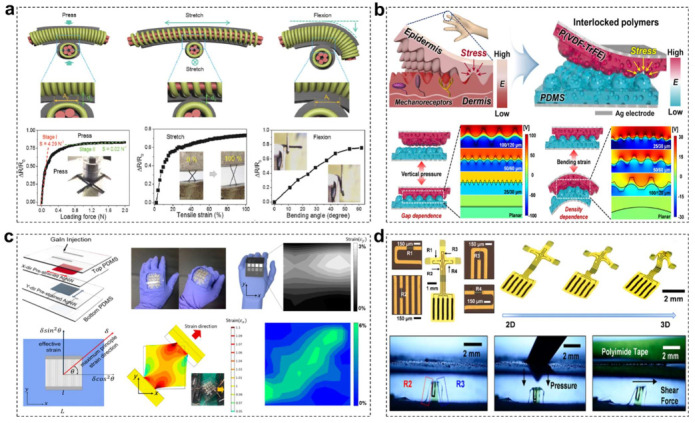
(**a**) Schematic illustration and relative resistance variation of electronic fabric artificial skin in response to pressure, stretching and flexion force. Reprinted with permission [48]. Copyright 2016, Wiley-VCH. (**b**) The epidermis-dermis layer with interlocked micro-ridges in human skin and the skin-inspired nanostructured e-skin. Schematic illustration and theoretical calculation of the gap distance change of the resultant electronic sensors under vertical pressure and bending strain. Reproduced with permission from ref. [50]. Copyright 2018 American Chemical Society. (**c**) Schematics of the multidimensional strain sensor with X-directional and Y-directional pre-strained AgNW. Demonstration of applications of multidimensional strain sensors to map the strain distribution for hand grip motion. Reprinted with permission from ref. [58] (Copyright 2015 American Chemical Society) (**d**) Optical images of 2D precursor that have two piezoresistors (R2 and R3) in the vertical direction and the other two (R1 and R4) in the horizontal direction, and their 3D structure evolutions during the mechanical buckling processes. The bottom images are the microscopic pictures of the as-fabricated sensor in response to normal force and shear force. Reprinted with permission [60]. Copyright 2019, American Chemical Society.

To address this challenge, many research groups have endeavored to develop wearable electronics with skin-mimicking multidirectional force sensing performance via diverse material design strategies. Firstly, one of the promising strategies is designing novel force-sensitive conductive architecture upon soft polymer substrates. For example, Yu and coworkers reported a stretchable electronic fabric consisting of intertwined composite fibers with the helical silver nanowires network as the conductive core electrode and piezoresistive rubber as a shell sensing element (Figure 1a) [48]. Thanks to the unique coaxial structure of the stretchable sensor electrode and fibrous sensing architecture, the resultant fabric sensor arrays could simultaneously map and quantify multiple mechanical stresses, including normal pressure, lateral strain and flexion. Zhu and coworkers demonstrated a structural engineering of gold thin films with channel cracks, which can greatly improve the response gauge factor to external subtle tensile and pressure force [49]. In addition to these unique structures, flexible electronics with biomimetic structure design are also capable of detecting multiple mechanical forces. In human skin, owing to the presence of stiff epidermis and soft dermis with micro-ridge structures and gradient stiffness, the applied stress could be effectively concentrated and amplified, and then be transferred to underlying mechanoreceptors for enhanced tactile sensing. Inspired by this special skin structure, Ko and coworkers proposed a skin-like hierarchical nanoporous and interlocked micro-ridge structured polymer architecture with gradient elastic modulus for the fabrication of highly sensitive triboelectric sensors (Figure 1b) [50]. Thanks to the bionic structure design, the resultant electronic sensors presented the maximized triboelectric charge generation and enhanced sensitivity to force, showing great potentials in detecting pressure, bending and other motions. Interestingly, as reported by other workers, different engineered microstructure geometries (i.e., dome, pyramid and pillar) between interlocked layers also have nonnegligible influence to the multidirectional-force detection properties [51,52]. Moreover, some other bionic engineering structures including fingertip, whiskers and microcrack have also been employed to explore more elaborate force-related detecting parameters (such as texture, roughness, and stiffness) [4,53,54]. Secondly, the direct integration of individual strain and pressure sensor is the simplest but efficient method to achieve multidirectional-force sensitivity in e-skin device. Nevertheless, if adopting this method, the response signal interference problem among each force sensor should be primarily considered. Bao and coworkers demonstrated a promising vertical stacked sensor strategy that could independently distinguish strain and pressure force [55]. The strain force was determined by the comb capacitance electrode sensor at bottom layer that is intrinsically sensitive to both strain and pressure, while the pressure sensor with the microstructure capacitor design only detected the vertical pressure force. This structure design effectively deduces the influence of horizontal strain to vertical pressure detection and minimize the signal interference.

On the other hand, accurately discriminating multidirectional force stimuli is highly desirable for e-skin, which could provide more concrete contact information for users, such as contact location, force direction and contact surface. These parameters are quite essential for developing a robotic equivalent of human skin and its complex sensory system. However, most reported force sensors have shown nearly uniform signal responding to multiple loading conditions, owing to the Poisson’s ration induced strong coupled electrical change in major axis of the principal strain and perpendicular direction [56,57]. This makes it difficult to efficiently detect and discriminate complex multiaxial and multidimensional stress conditions, causing the feasible application limited to only simple human motions such as finger or joint bending. Recent research efforts have been devoted to attempting to overcome this limitation. Ko and coworkers reported a multidimensional strain sensor consisting of two pre-strained anisotropic Ag NW percolation network layers intersecting each other, showing the capacity to simultaneously detect and discriminate multidimensional strain loadings (Figure 1c) [58]. By means of decoupling electrical resistance change to the major axis of the principal strain and perpendicular direction, the resultant electronic sensor could independently detect the component force in X and Y axes direction, thereby obtaining complete resolution of the surface environment. The proposed Ag NW network was further utilized to construct a 4 × 4 multipixel strain sensor array via ultraviolet (UV) laser ablation, allowing various strain conditions to be simultaneously monitored and modeled by surface strain distribution mapping. Although this sensor has distinct advantages in discriminating 2-dimentional (2D) in-plane force, it is still a challenge for them to detect and distinguish random 3-dimentinal (3D) out-plane force.

The design and manufacture of electronic sensors with a 3D spatial response structure provide an alternative method to solve this obstacle. Compared with traditional 2D planar sensor, 3D sensors have one more degree of freedom in Z axis direction, enabling them precisely discriminate complex stress from all spatial directions. Bao and coworkers developed a biomimetic soft capacitive-type tactile sensor, highlighting the superb capacity to detect the direction of applied force in real time [59]. It was enabled by a 3D structure that mimics the interlocked dermis-epidermis interface in human skin. The top layer of the e-skin comprised a grid of molded square pyramids, while the bottom layer of the e-skin comprised a 2D array of molded hills. When applying a tilt force to the e-skin, the capacitors locating on the side of the hill exposed to a greater pressure and had a larger increase in capacitance than those located on the side opposite the applied force direction. The obtained capacitance map around a hill provided the information to differentiate normal force, shear and tilt force, illustrating its potential application in robotics with spatial tactile feedback. Moreover, Rogers and coworkers reported a unique 3D piezoresistive structure that endows microelectromechanical sensors with separating and decoupling multidirectional force stimuli (Figure 1d) [60]. The 2D planar precursor incorporated four silicon nanomembranes (Si-NM) elements at strategic locations across a patterned thin polymer film. After mechanically guiding the geometry transformation of the precursor, a 3D electronic sensor with a table-like shape (planar top surface with four supporting legs) came into being. The resulting material system could accurately detect and separate in-plane and out-of-plane mechanical deformations through independent electrical interfaces to these four piezoresistive Si-NM elements, enabling the simultaneous recording of pressure, shear force, and bending strain, along with temperature. The schemes extended naturally to arrays of devices for spatiotemporal mapping of responses, which may provide wide utility in robotic interfaces, prosthetic control systems, and medical diagnostics.

### 2.2. Multiple Physical Chemistry-Stimuli Sensing Materials

Physical chemistry parameters, mainly including temperature and humidity, are important indicators for both external environmental change and inner human health condition. Body temperature, as a basic physiological index, plays an indispensable role in many medical symptomatic diagnose, such as fever, pneumonia, infection or influenza [10]. The emerging wearable temperature sensors, conformally mounted on human skins with minimal user awareness, make it possible to continuously monitor temperature variation and timely discover abnormal signal change based on responding signal feedback. Apart from the temperature, humidity is the other useful physical chemistry parameter. It also provides vital physiological information for human health, which has certain correlation with some respiratory and lung diseases [9]. Generally, the exhaled air from human nose contains more moisture than the inhaled air. By means of detecting moisture content of the air near the nose during human breathing process, one can judge whether the person is breathing normally. Therefore, developing electronic sensors with physical-chemistry-parameters detecting capacity not only promotes the exploitation of multifunctional artificial e-skin materials, but also contributes to extending their practical application in healthcare and environment monitoring fields.

**Figure 2:**
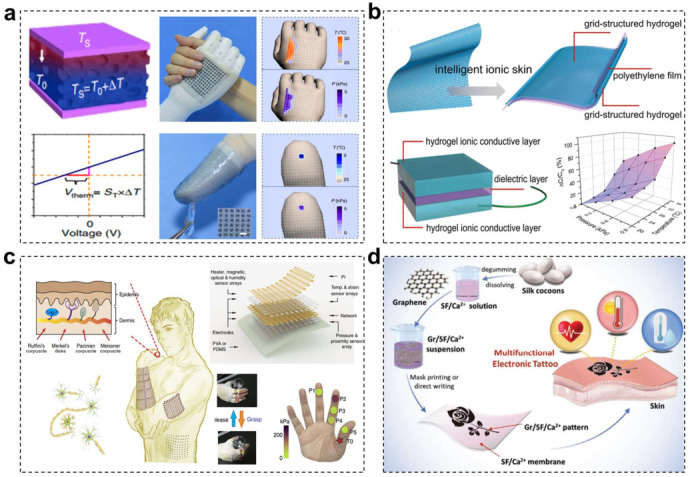
(**a**) Schematic illustration of sensing mechanism under a coupled temperature and pressure stimuli. Digital photos of a prosthetic hand arm in contact with an adult woman and an e-finger touching an ice cube. The graphs display the corresponding temperature and pressure mapping signal profiles of the prosthetic hand. Reprinted with permission from ref. [75]. Copyright 2015 Springer Nature. (**b**) Schematic illustration of an ionic skin fabricated from the printed hydrogels and a capacitive sensor device. The corresponding capacitive response of the hydrogel sensor upon applying pressure and temperature. Reprinted with permission from ref. [80]. Copyright 2017 Royal Society of Chemistry. (**c**) Schematic illustration of SCMN conforming to the surface of human skins. Schematic layout of an integrated sensor array with eight functions. The images show the operations of the intelligent prosthetic hand to grasp and release water and the corresponding sensor distribution. Reprinted with permission from ref. [95]. Copyright 2018, Springer Nature. (**d**) Schematic illustration of the fabrication process of multifunctional Gr/SF/Ca^2+^ E-tattoo. Reproduced with permission [97]. Copyright 2019, Wiley-VCH.

Benefiting from the piezoelectric and pyroelectric attributes, human skin is not only able to detect various physical force stimuli such as tactile and tensile stress, but also has superb capacities to monitor some physical chemistry stimuli. Among these biomimicking multiple-stimuli perception, wearable electronic devices with skin-like force and temperature perceptual abilities have attracted the most extensive concerns, which shows great potentials in robotic, prosthetics and healthcare applications [61,62,63,64,65]. Specifically, graphene, CNTs, PANI nanoparticles, Ag nanocrystals, stretchable metal and Si nanoribbons with special structure design (such as serpentine, wrinkle, net-shaped) are widely chosen as the promising thermosensitive elements for multifunctional e-skin devices [66,67,68,69]. Metal-based thermoresistive sensors usually exhibit an increase in resistivity with increasing temperature due to the decrease in electrons mobility, while semiconducting and charge hopping dominant materials undergo decrease in resistivity on account of the increased charge carrier density or increased thermally assisted charge carrier hopping, respectively. Making full use of these flexible thermo-responsive sensing materials and structural engineering design, various dual-parameter temperature-pressure sensors for different application scenarios have been demonstrated in the past few decades [70,71,72,73,74]. For example, Zhu and coworkers reported a flexible dual-mode temperature and pressure sensor based on microstructured-frame-supported organic thermoelectric (MFSOTE) materials (Figure 2a) [75]. Taking advantage of independent thermoelectric and piezoresistive effects in a single MFSOTE device, simultaneously monitoring temperature and pressure was realized by transducing external stimuli into separate electrical signals. Notably, the devices can be self-powered by a natural temperature gradient with a high-temperature detection resolution and pressure-responsive sensitivity of up to 0.1 K and 28.9 kPa^−1^, respectively. Someya and coworkers demonstrated a gas-permeable, inflammation-free, lightweight, stretchable on-skin electronics realized with a conductive nanomesh structure [76]. The as-prepared electronic sensor can directly laminate onto human skin for long-time temperature, pressure and touch detection with minimal invasiveness, which shows promising application in the clinical healthcare field. In addition, inspired by the sophisticated sensory structure in human fingertips, Ko and coworkers fabricated a multimodal e-skin based on flexible and micro-structured ferroelectric films composed of poly(vinylidene fluoride) and reduced graphene oxide (rGO) [77]. Thanks to the bionic structure design, the as-prepared e-skin showed improved sensing performance for both spatiotemporal tactile stimuli (static and dynamic pressure) and temperature.

Furthermore, different from above e-skin materials generally composed of flexible elastomer substrate and functional conductive fillers, hydrogel materials with water-infiltrated microporous structure become another type of alternative constitute materials for wearable electronics and bionic devices. They have similar physiological and mechanical properties to human skin, and possess many obvious advantages over traditional sensing materials, such as independent ionic conductivity, desirable biocompatibility, excellent adaptivity, good transparency and stretchability. Hence, many research groups have endeavored to develop hydrogel-based electronics with skin-like multiple sensations [78,79]. Wu and coworkers proposed a feasible strategy that incorporates stimuli-responsive micro-structured hydrogel into capacitor circuit to prepare multifunctional e-skin materials (Figure 2b) [80]. The 3D printed intelligent skin exhibited a temperature sensitive behavior and a high pressure-sensitivity of 1 kPa, allowing it to detect body temperature, gentle finger touches and finger bending motion sensitively. They also developed a type of biomimetic iontronics to imitate natural skins using supramolecular polyelectrolyte hydrogels [81]. The dynamic viscoelastic networks provided the biomimetic skin with a wide spectrum of mechanical properties and polyelectrolytes’ ionic conductivity allows multiple sensory capabilities toward temperature, strain, and stress. Recently, Wu and coworkers utilized a facile salt-percolated strategy to fabricate hydrogel-based temperature and strain sensors with excellent freezing and drying tolerances [82]. The resultant sensors exhibited high thermal sensitivity, wide temperature detection range, low strain detection limit, short response time, and low hysteresis, making it attractive for next-generation wearable electronics.

As for humidity sensing, the common transducing mechanism is that a higher water content normally gives rise to a better conductance due to its intrinsic ionic conductivity. The introduction of moisture-responsive sensing elements or hydrophilic substrates in electronic sensor is a frequently adopted method to endow wearable e-skin with humidity perceiving ability [83,84,85,86]. For example, hygroscopic celluloses could reversibly adsorb water from the surrounding environment and become the ideal flexible substrate for humidity sensor. Whitesides and coworkers reported an effective and simple method to prepare a digitally printed paper-based moisture sensor for monitoring respiration [87]. Moreover, other conductive materials with hydrophilic functional groups, such as polyaniline, silicon nanocrystal, modified graphene and CNTs hybrids, could also generate resistance change responding to external moisture change [88,89]. Thereby, this type of conductive materials has been widely chosen as the moisture-response element for humidity sensing. Although tremendous efforts have been made to develop single moisture responding sensor with high sensitivity, the research works about electronic sensors which would simultaneously detect force-moisture dual stimuli are relatively less. Our group reported a multifunctional self-healable electronic sensor integrating strain and humidity detecting ability in one single material system [90]. This was enabled by a unique strain-dependent conductive microcrack material consisting of hydrophilic cellulose nanocrystals (CNC) and CNTs nanohybrids (CNC@CNTs). Under tensile stress, the size of microcrack wound gradually broadens, resulting in an increased resistance response. In addition, the conductivity of the CNC@CNTs layer would greatly increase when exposed to environmental moisture. Liu and coworkers demonstrated a novel artificial ionic skin comprising with a bilayer of oppositely-charged, double-network hydrogel [91]. The ionic sensor can convert both mechanical and humidity stimuli into four types of electrical signal variation (resistance, capacitance, open circuit voltage and short circuit current) based on stimulus-controlled ion mobility, which are prospective sensing materials in soft robotics, wound dressing wearable and implantable devices.

Complicated application scenarios with manifold environmental factor changes put forward more stringent demands for the sensory ability of wearable e-skin. More than dual parameters monitoring, flexible electronic sensors with ability to simultaneously perceive and discriminate multiple external stimuli are receiving more and more attentions. This could be realized by the direct integration of multiple stimuli-responsive sensors [92,93,94]. Wang and coworkers reported a stretchable and conformable matrix network (SCMN) as multi-sensory e-skin integrating actualized specific expandable sensors on a structured polyimide network (Figure 2c) [95]. It exhibited multiple sensory capabilities including but not limited to temperature, in-plane strain, relative humidity (RH), UV light, magnetic field, pressure, and proximity. Kim and coworkers demonstrated a stretchable silicon nanoribbon electronics for smart prosthetic skin. By means of equipping with different stimulus-responding sensor arrays, the e-skin would efficiently detect temperature, press, strain, and humidity [96]. However, large-area integration of the multiple sensors with different sensing principles in a pixel of the multimodal e-skin requires sophisticated fabrication processes. Moreover, apart from sensor integration strategy, multi-sensory e-skin could also be constructed in one single material system based on some special multi-stimuli-response substances. For example, Zhang and coworkers reported a healable and multifunctional E-tattoo based on a graphene/silk fibroin/Ca^2+^ (Gr/SF/Ca^2+^) combination (Figure 2d) [97]. The graphene flakes distributed in the matrix formed an electrically conductive path that is responsive to environmental changes, such as strain, humidity, and temperature variations, endowing the E-tattoo with high sensitivity to multi-stimuli. Wu and coworkers developed a special adaptive polyionic elastomer composing of an amorphous long-chain polyanion (polyacrylic acid, PAA) and a polycation (poly(methyl chloride quaternized *N*,*N*-dimethylamino ethylacrylate)) [98]. The polyionic elastomers were highly transparent, 3D-printable, ultra-stretchable, self-healable, self-powered, and capable of sensing strain, stress, touch, humidity, temperature, etc. Zhang and coworkers prepared a multifunctional nanosensor consisting of ZnO nanowires and polyurethane (PU) fibers, which combined high stretchability (>50% strain) with three different sensing capabilities, i.e., strain, temperature, and UV [99].

Despite brilliant achievements in multimodal perception, the response signal interference problem toward different stimuli is still a non-negligible barrier for the development of e-skin devices. Specifically, in flexible multimodal electronic sensors, the target signal variation responding to specific stimulus tend to be influenced by the other environmental parameters owing to their multi-stimuli responsiveness of sensing elements. This similar disturbance phenomenon also exists in human skin, causing the inaccurate perception and discrimination under multiple stimuli. When two coins with same weight but different temperatures are attached to human skin, we erroneously perceive that the colder coin is heavier than the warmer one, owing to the slow-adapting mechanoreceptors in the skin respond to thermal and pressure stimuli. To address this problem, some research groups minimized the signal interference by means of designing compensating circuits and reference devices, which would simply calibrate the output signals [100,101,102]. However, this approach had only limited success due to the design of sophisticated circuit, the complex fabrication process and increased costs. The utilization or designing sensing materials with selective stimuli sensitivity provide feasible solutions to eliminate signal coupling. Cho and coworkers reported a pressure/temperature bimodal electronic skin with linear sensitivity and near-perfect stimulus discriminability [103]. A high pressure-sensitivity with negligible temperature dependence was obtained by means of employing temperature-independent Al_2_O_3_ with high dielectric constant as dielectric layer. Temperature sensing without pressure dependence was realized via introducing a novel nonphysical-contact design for temperature-sensing rGO. Another alternative approach is measuring multiplex signals based on disparate sensing mechanism in one single electronic device. Recently, Wang and coworkers reported a hierarchically patterned self-powered sensor for multifunctional tactile sensing, consisting of hydrophobic films and graphene/Polydimethylsiloxane (PDMS) sponges [104]. Based on the piezoresistive and thermoelectric effects of graphene/PDMS sponge, the electronic device showed a high temperature detection resolution of 1 K and pressure-sensing sensitivity of 15.22 kPa^−1^ according to different output response signals. The most interesting result is that the resultant sensor would infer diverse material properties according to the electrical signals generated between the PTFE film and objects through the triboelectric effect.

### 2.3. Multiple Biochemistry-Stimulus Sensing Materials

Flexible biochemical sensors, which could rapidly detect biomarkers from human biofluids, become effective tools to monitor the human health in real time and give early diseases diagnosis. Different from above-mentioned electronic sensors that focus on monitoring the physical activities and vital environmental signals, biochemical sensors could provide the individual’s health information at molecular levels in a more straightforward and noninvasive manner. Over the past few decades, tremendous progress has been made to fabricate wearable biochemical sensors with the abilities to continuously and timely monitor diversity of biochemical signals, such as analytes, metabolites (e.g., glucose, lactate, uric acid and creatinine), electrolytes (e.g., Na^+^, K^+^, Ca^2+^, NH^+^, Cl^−^, pH), heavy metals (e.g., Cd^2+^, Pb^2+^, Cu^2+^, Zn^2+^, Hg^+^) and some intake substances (caffeine, alcohol and cortisol) [105,106,107]. Moreover, various biofluid that collected from human body have been applied as the main carriers of these biomarkers, mainly including sweat, blood, saliva, tears and urine. Compared with other biofluids, human sweat is the most widely studied one for noninvasive analysis, which could be gathered and tested noninvasively, painlessly, continuously and conveniently. It also contains abundant important biochemical markers, such as glucose, ethanol, lactate, electrolytes, etc., which reveal the body’s physiological state [108,109]. For example, glucose is the human body’s energy source and long-time glucose dysregulations may increase the illness risk of obesity or type II diabetes mellitus. Lactate is the generated product of glucose during anaerobic metabolism and an increase of lactate level may be associated with diseases such as endotoxic shock, sepsis, intestinal infarction, cardiac arrest and resuscitation. Electrolytes play a key role in maintain the cellular fluid balance and include Na^+^, K^+^, Ca^2+^, Mg^2+^, Cl^−^ ions, phosphate, bicarbonate. The imbalance of these electrolytes has negative influence on human body, which is highly related with biofluid buffering, fluid movement regulation, membrane permeability, nerve excitability and endocrine secretion [2,9,10]. Therefore, the accurate, sensitive, real-time, continuous and noninvasive monitoring and analysis of bio-signal molecules in human sweat is of paramount significance for the early diagnostics and prevention of relevant diseases. Wearable biochemical sensor technology provides promising potentials to unobtrusively monitor biological parameters reflecting the physiological state of human body.

First of all, the sweat generation and sampling should be taken into primary consideration, which supply basic testing specimen for sweat analysis [110]. Previous sweat sensors rely on vigorous physical exercise or exposure to heat to extract a certain amount of sweat from human skin. However, these perspiration methods are inconvenient and the composition of as-produced sweat could not be representative of the true health condition of human body. To address this problem, iontophoresis has been specially exploited to obtain local and on-demand sweat naturally via adopting a sweat-inducing compound called pilocarpine [111]. After applying a mild electric current, the pilocarpine would release into to the skin and stimulate the sweat glands to secrete sweat. This noninvasive and safe sweat generation technique has been approved by the Food and Drug Administration and frequently adopted by most microfluidic-based sweat biochemical sensor. Moreover, reverse iontophoresis with similar mechanism is also developed to collect interstitial fluid without the requirement of drug. When stimulate the epidermis by the electric current, some chemicals in interstitial fluid would migrate to the skin surface according to electro-osmotic effect. Then, the as-acquired sweat or interstitial fluid would fill the microchannel of sweat-based sensing device or be gathered in micro-reservoirs for the next analysis and detection process.

**Figure 3:**
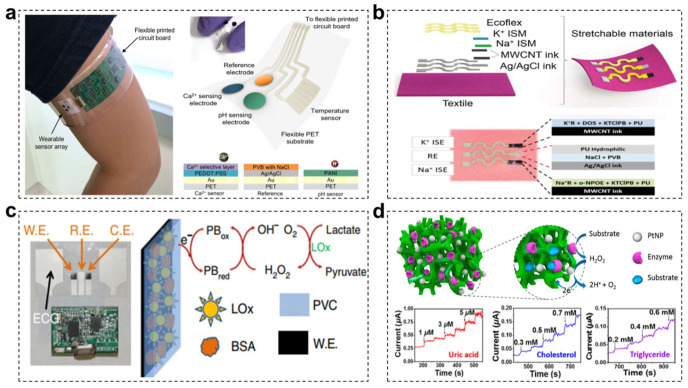
(**a**) The fully integrated wearable multiplexed sensing system on a subject’s arm. Diagrams of a flexible sensor array containing Ca^2+^, pH, and temperature sensors patterned on a flexible polyethylene terephthalate substrate and corresponding surface membrane electrode compositions. Reprinted with permission from ref. [113]. Copyright 2016 American Chemical Society. (**b**) Schematic representation of the tailor-made stretchable materials and the wearable sensor based on textile and ion-selective membranes composition. Reproduced with permission [114]. Copyright 2016, Wiley-VCH. (**c**) The images of a Chem-Phys patch along with the wireless electronics. The schematics show the lactate oxidase-based lactate biosensor along with the enzymatic and detection reactions. Reproduced with permission [116]. Copyright 2016, Springer Nature. (**d**) Schematic illustration of the general sensing mechanism of the resultant hydrogel electrode platform based on PANI/PtNPs/enzyme hybrid films. Reprinted with permission from ref. [117]. Copyright 2015 American Chemical Society.

Biochemical sensor is the core component of wearable sweat-based sensing device, which could efficiently generate electric signal variation in response to the target analytes. Owing to the low concentrations and contents of chemicals in human biofluids, the practically applicable biochemical sensors should be equipped with high sensitivity, low detection limit, specific selectivity, wide detection range as well as desirable repeatability. In order to meet these strict requirements, different kinds of biochemical sensors have been specially developed, mainly including electrochemical devices, chemiresistors and transistors [112]. Among them, electrochemical sensors have been widely used in wearable e-skin due to their features of simple operation, convenient integration, easy miniaturization, rapid and label-free detection. According to different working principles, electrochemical sensors could be divided to potentiometric and amperometric sensors [10]. They are primarily composed of reference, working and auxiliary electrodes. Potentiometric sensors generally adopt ion-selective electrodes or membranes to selectively generate potential changes responding to target analytes, especially charged species (such as Na^+^, NH^4+^, K^+^ ions and protons) in sweat. Since the electric potential of an ion-selective electrode strongly depends on the target analyte concentration, the analyte concentration can be deduced via figuring out the potential difference between working and reference electrodes. For example, Javey and coworkers prepared a wearable electrochemical platform for noninvasive and continuous analysis of Ca^2+^ ion and pH in body fluid (Figure 3a) [113]. In this work, ion-selective electrodes were utilized to measure Ca^2+^ concentration and pH, accompanied with using polyvinyl butyral-coated Ag/AgCl as the reference electrode. The Ca^2+^ concentration and pH value deduced from the electric potential difference were consistent with these detected by commercially available testing technique, which were conducive to the primary hyperparathyroidism and kidney stones disease diagnosis. In addition, taking advantages of ion-selective mechanism, multiple-ion detection would be realized via the integration of different ion-selective electrode. For example, wang and coworkers fabricated a stretchable textile-based multi-ion potentiometric sensor using ion-selective membranes to selectively measure Na^+^ and K^+^ with a wide detection range (Figure 3b) [114]. A wearable sweat analysis platform integrated with the electrochemical enhanced iontophoresis interface and two selective electrodes was developed by Davis and coworkers to real-time monitor glucose, Na^+^ and Cl^−^ for the cystic fibrosis diagnosis [115]. However, these potentiometric biosensors are susceptible to interference from other charges for less concentrated ions detection and strongly rely on the ion-selective membrane layer.

As for amperometric sensors, enzymes-immobilized electrode is chosen as the working electrode, which could selectively catalyze the oxidation/reduction reaction of target redox-active biomolecules (such as glucose, lactate, alcohol and caffeine) and thereby produce electric current variation in response to changing analyte composition. Conductive polymers (e.g., PANI, PPY, PEDOT), carbon-based nanomaterials (e.g., CNTs, graphenes) and some metal-based nanoscale materials (e.g., Ag, ZnO) are commonly used electrode materials for enzyme immobilization and biosensing due to their excellent biocompatibility, unique electrochemical activity and desirable flexibility. Benefitting from the intrinsic specificity of different enzymes, these amperometric devices possess high selectivity and excellent catalytic ability for diverse substance. For example, Mercier and coworkers demonstrated a wearable chemical-electrophysiological hybrid biosensing system for simultaneously monitoring lactate and electrocardiogram (Figure 3c) [113]. The immobilized lactate enzyme catalyzed the oxidation of lactate to generate pyruvate and H_2_O_2_ once exposure to the lactate in sweat. Then, the generated H_2_O_2_ would be selectively reduced by Prussian blue transducer and generate electrons to induce current change. The increase in current was correlated to lactate concentration in sweater. Moreover, various metabolites could be simultaneously detected in one single biosensor system by means of integrating different enzymes into each electrode for the respective detection with minimum crosstalk. Yu and coworkers reported a nanostructured conductive hydrogels-based biosensor platform for detecting diverse human metabolites (Figure 3d) [114]. By means of immobilizing different enzymes in PANI and Pt nanoparticles (PtNPs) electrodes, the as-prepared biosensors exhibited high sensitivity and wide detection range for uric acid, cholesterol and triglycerides, as well as low sensing limit. Despite the high efficiency of enzyme-based biochemical sensors for metabolite detection, they also have disadvantages such as the high cost of enzymes, complex immobilization processes, and poor stability.

On the other hand, most reported biochemical sensors for healthcare application focused on improving the sensing ability of single physiological signals. Given that one illness is always accompanied with the anomalism of multiple indicators, it is not sufficient and accurate to diagnose a disease and assess comprehensive health condition. Hence, developing wearable e-skin that could simultaneously detect various physiological signals is highly sought after for both individual health monitoring and personalized diagnosis [118,119,120,121]. As mentioned above, simultaneous and selective detection of different metabolites or ions could be achieved via employing different ion-selective electrodes or enzymes in potentiometric and amperometric sensor. These multi-analyte sensors based on electrochemical electrodes are easy to integrate and fabricate due to their similar sensing mechanism and device structure design. However, there are still huge challenges to integrate various sensors with multivariate mechanisms in one single wearable device, which require to synchronously solve multiple signal interference and scalable processing fabrication problem. To overcome these difficulties, Kim and coworkers reported a wearable patch for sweat-based diabetes monitoring and feedback therapy based on an efficient electrochemical interface consisting of a serpentine bilayer of gold mesh and gold-doped graphene [122]. By means of incorporating with a heater, temperature, humidity, glucose and pH sensors along with polymeric microneedles, the patch could be thermally activated to deliver Metformin transcutaneously and reduce blood glucose levels once the as-measured glucose value exceeds standard. It should be noted that the presence of humidity, temperature and pH sensors not only provided external environmental information, but also efficiently calibrated the glucose reading. Moreover, a light-weight, battery-free, skin-interfaced microfluidic/electronic system for sweat analysis was fabricated by Rogers and coworkers via integrating chronometric microfluidic platforms with embedded colorimetric assays [123]. This sensing platform combined electrochemical, colorimetric and volumetric analysis modes, which realize simultaneous and noninvasive detection of glucose, lactate, chloride, pH, and sweat rate/loss. Furthermore, Javey and co-workers demonstrated a wearable smart wristband consisting of fully integrated sensor arrays for multiplexed in situ perspiration analysis [124]. The resultant sensor platform was capable of simultaneously monitoring multiple physiological signals, including sweat metabolites (glucose and lactate), electrolytes (Na^+^ and K^+^ ions) and skin temperature (correct the response of these sensors). Making use of wireless transmission technology, all necessary information could be transferred to portable devices and cloud servers, which exhibits great potentials in real-time assessment of human physiological state.

## 3. Strategies for E-Skin with Self-Healing Abilities

Thanks to the autonomous ability to repair wounds, biological skins can recover their original appearance and critical functions after physical damage. Inspired by this unique self-repairing attribute of biological skin, advanced e-skins are equipped with the self-healing ability to extend their service life and reduce maintenance costs. In order to achieve satisfying self-recovering property, self-healable polymeric materials are specially designed and applied as the core functional component for self-healing electronics [18]. According to different healing mechanism, they could be generally categorized into extrinsic and intrinsic self-healing materials [125,126,127]. The former one is constructed by means of encapsulating healing agents in the capsules or vascular network of polymer substrate. When encountering evitable damage, theses healing agents would be automatically released at the damaged regions and trigger the polymeric polymerization or crosslinking reaction to self-repair the fractures. Although this strategy achieves a high-efficiency healing process, the limited healing cycle and complicated fabrication procedures restrict its practical application in flexible e-skin. To address these problems, intrinsic self-healing materials are consequently developed, which would autonomously recover the pristine mechanical and functional properties after repeated damage. This is enabled by designing noncovalent or dynamic covalent interactions with reversible attributes in polymer matrix, including hydrogen, disulfide, dynamic boroxine bonds, metal-ligand coordination, ionic interactions and π-π interactions. Once the self-healable materials occur mechanical breaking, polymer chains with low glass transition temperature and high mobility would quickly diffuse into the damaged areas and reconstruct the dissociated dynamic bonding linkage, which efficiently facilitate restore their original properties. Owing to their structural simplicity and healing repeatability, intrinsically self-healing systems are frequently adopted to fabricate self-recovering electronic sensors, supercapacitors, electrodes, semiconductor, cells and actuators [125,126,127,128,129,130,131,132,133,134,135]. In this section, we will discuss recent advances in the design strategy of self-healing materials and their potential applications in wearable e-skin devices.

### 3.1. Single Dynamic Crosslinking Network

**Figure 4:**
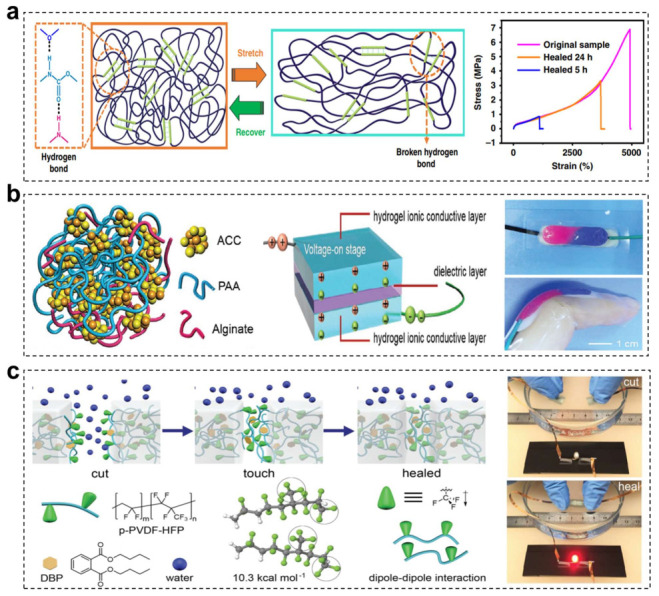
(**a**) Schematic illustration of the breaking and reforming of hydrogen bonding that resulting in high stretchability and desirable self-healing property. Stress-strain behavior of the self-healed PUA film with different healing time at 100 °C. Reprinted with permission from ref. [143], Copyright 2019 Springer Nature. (**b**) Schematic structure of the amorphous calcium carbonate/ polyacrylic acid/alginate mineral hydrogel based on the dynamic chelation of the Ca^2+^ and carboxylic groups, and the structural design of ionic skin. Photos of the autonomously healed hydrogel sensor and the self-healing hydrogel sensor attached to a bent finger. Reproduced with permission [144]. Copyright 2017, Wiley-VCH. (**c**) Underwater healing process of highly polar and hydrophobic fluorinated polymers with dynamic dipole-dipole interactions. The photos of completely severed conductor and electrical conductivity recovered immediately when contacted. Reproduced with permission [147]. Copyright 2018, Wiley-VCH.

Dynamic polymeric materials with single dynamic crosslinking network are typical self-healing matrixes for flexible electronics. Different single reversible bonds with respective characteristic as-mentioned above are utilized to dynamically crosslink polymer materials and endow them with autonomous self-healing capabilities [136,137,138,139,140,141]. In 2012, Bao and coworkers pioneered an electrically and mechanically self-healing composite material composed of nanostructured nickel microparticles embedded in a supramolecular hydrogen-crosslinked polymer [142]. Taking advantages of the dynamic nature of the urea-based hydrogen bonds, the resultant composites with lower glass transition temperature exhibited outstanding electrical healing efficiency (~90%) in 15 s and complete recovery of mechanical properties in about 10 min under ambient temperature when occur mechanical damages. Then, a self-healing electronic skin with pressure and flexion sensing abilities was delicately fabricated based on this conducting supramolecular polymeric composite. Despite desirable self-healing efficiency, this material based on single hydrogen bonded network is limited to the relatively low mechanical properties. Lee and coworkers demonstrated an extremely stretchable and self-healing conductor via embedding liquid metal and silver flakes into supramolecular polyurethane acrylate (PUA) matrix (Figure 4a) [143]. With the supramolecular hydrogen bonding design, the elastomer exhibited a high tensile strength of nearly 7 MPa, ultra-high elongation at break (5000% strain) and desirable self-healing efficiency. After blending with conductive fillers, the resultant conductor could self-recover 96% of its original conductivity (6250 S cm^−1^) after mechanical breaks. This self-healable thermoplastic elastomer composite showed great potentials in triboelectric nanogenerator and soft electronic devices application. Although the elastomer possessed high original mechanical properties, the higher healing temperature (100 °C) and longer healing time were required to achieve satisfactory recovery performance. Besides single dynamic hydrogen bonding network, other dynamic crosslinking networks are also frequently introduced to self-healing system, such as ionic bonds, metal-ligand coordination bonding, dynamic covalent bonds, ion-dipole interactions and so on. For example, Wu and coworkers fabricated a self-healable mechanically adaptable ionic skin based on a supramolecular mineral hydrogel for highly sensitive pressure sensing (Figure 4b) [144]. Specifically, a small amount of amorphous calcium carbonate nanoparticles was utilized to physically crosslinked PAA and alginate chains, which served as the basic material of self-healable ionic skin. Based on the dynamic chelation of Ca^2+^ and carboxylic groups, the as-prepared material could autonomously self-heal within 20 min at ambient condition and exhibited a recovery efficiency of 82%. Moreover, Yu and coworkers reported a conductive self-healing hybrid gel by mean of designing metal-ligand supramolecular bonding in nanostructured conductive supramolecular gel [145]. In this material, the nanostructured PPY gel was adopted to construct a conductive network and the supramolecular gel played a role of self-healing elements in the hybrid gel. The as-prepared hydrogel showed high electrical conductivity (12 S m^−1^), excellent self-recovering properties at room temperature and enhanced mechanical strength, which could be utilized as fundamental matrix for self-healable electrical circuit.

Moreover, developing flexible e-skins with underwater self-healing performance are highly attractive for their applications in aquatic or marine environments, such as water-resistant human-machine interfaces and aquatic soft robots. Previous reported self-healing materials based on hydrogen bonds or metal-ligand coordination are not suitable for operating under aquatic conditions, because water molecules can act as hydrogen donor/ acceptor, ligands and polar solvents to form new noncovalent bonds and disrupt the original bonding composition. As a result, such self-healing materials will swell and lose self-healing ability in aqueous environments. To overcome this disadvantages, Tee and coworkers reported a novel material termed “GLASSES” via combining amorphous polymer with ionic species to fabricate aquatic and self-healing stretchable electronic skin [146]. “GLASSES” consisted of a highly polar fluoro-elastomer and a fluorine-rich ionic liquid and they would interact with each other to form reversible ion-dipole interactions. Both two components were hydrophobic and had weak interactions with water molecules. Consequently, water environments would minimally perturb the dynamic ion-dipole interactions, making GLASSES materials available to self-heal under aquatic environments. The GLASSES resultant composites were transparent, conductive and self-healable in dry, wet, acidic and alkali environment, which showed great potentials in emerging e-skin for unobtrusive underwater exploration. Furthermore, Wang and coworkers introduced a new dynamic dipole-dipole interaction in highly polar and hydrophobic fluorinated polymers to obtain underwater self-healing elastomers (Figure 4c) [147]. Taking advantages of this underwater self-healing elastomer, a self-healable conductor was fabricated via incorporating silver flakes with this substrate, which possess excellent electronic-mechanical self-healing abilities in air, underwater and harsh aqueous condition.

### 3.2. Multiple Dynamic Crosslinking Networks

Different dynamic supramolecular bonds have respective bonding characteristics and contribute to disparate healing effect. Self-healing materials based on supramolecular hydrogen-bonding crosslinking usually exhibit fast healing time and high healing efficiency, while their mechanical properties are relatively poor due to the low hydrogen bonding energy. Triple, quadruple hydrogen bonding, dynamic covalent bonds or metal metal-ligand coordination interactions design are beneficial to improve the mechanical performance of self-healing materials, but sacrificing healing time and remediation efficiency, and even requiring more strict healing condition such as heat, light or chemicals. Designing multiple supramolecular crosslinking networks in self-healing system integrates different self-healing attributes in one single material and achieve customized healing effect for diverse specific application scenarios. This strategy has gained increasing attention owing to its good structural designability and programmable self-healing effect.

**Figure 5:**
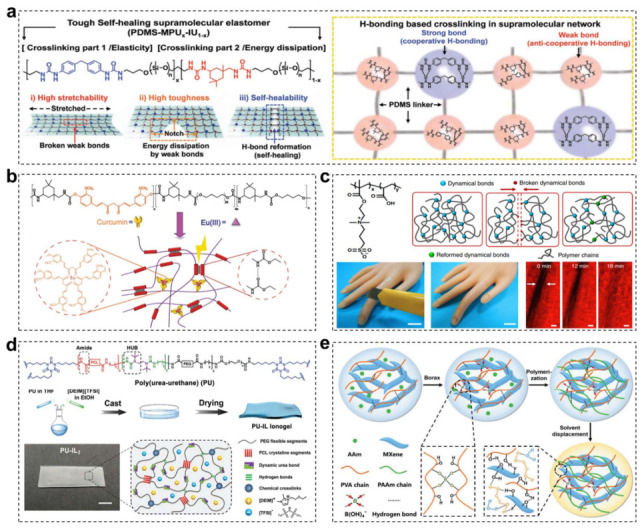
(**a**) Chemical structure of the supramolecular elastomer and possible hydrogen bonding combinations for strong bond and weak bond, respectively. Schematics of a stretched polymer film, a notched film, and a healed film. Reproduced with permission [149]. Copyright 2018, Wiley-VCH. (**b**) Molecular structure of curcumin (Cur)-embedded polymer. A schematic depiction of Eu (III) coordinated P-Cur material, highlighting the Eu-curcumin coordination bonds and the hydrogen bond derived from the urethane groups. Reproduced with permission [153]. Copyright 2018, Wiley-VCH. (**c**) The chemical structure of the polyelectrolyte and the schematic illustration of the dynamic process. Photos of a biomimetic skin whose hydrogel layer is cut into half and the healed biomimetic skin. Direct observation of the reconfiguration and autonomous self-healing of the dynamic networks by time-resolved fluorescence microscopic images (scale bar: 20 μm). Reprinted with permission from ref. [81], Copyright 2018 Springer Nature. (**d**) Chemical structure of the PU network and schematic of the fabrication process of PU-IL ionogels. Digital image of PU-IL2 ionogel and its corresponding schematic structure. Reproduced with permission [157]. Copyright 2020, Wiley-VCH. (**e**) Schematic illustration of the fabrication of a conductive, anti-freezing, and self-healing organohydrogel-based strain sensor with dual dynamic crosslinking networks. Reproduced with permission [159]. Copyright 2019, Wiley-VCH.

One of the promising principles for designing multiple supramolecular networks is the adoption of multiple supramolecular interactions with the same type, such as different hydrogen bonding or metal-ligand coordination [148]. For example, Bao and coworkers reported a tough and water-insensitive self-healing elastomer through rationally designed multi-strength hydrogen bonding interactions (Figure 5a) [149]. In this multiple supramolecular crosslinking system, the strong hydrogen bonds (4, 4′-methylenebis(phenyl urea) unit) enabled robustness and elasticity, whereas the weak bonds (isophorone bisurea unit) contributed to energy dissipation via reversible H-bonding breakage and reconstruction. This unique chemical structure design allowed resultant elastomer to exhibit notch-insensitive high stretchability, high toughness and autonomous self-healing even in artificial sweat. By means of incorporating EGaIn (liquid metal) alloy with this self-healable polymeric matrix, a stretchable and self-healing strain sensor could be obtained, which was able to detect diverse human motions and withstand undesirable damages. Furthermore, taking advantages of the same elastomer substrate, they developed an integrated self-healable electronic skin system via embedding conductive CNTs network on the top surface of the elastic film [150]. Interestingly, they found that once a conductive nanostructured conductive network was surrounded by a self-healing polymer substrate, the broken conductive network would follow the dynamic reconstruction of the self-healable polymer chains and autonomously heal to regain both high conductivity and mechanical properties. Based on this discovery, the conductive network could function as self-healable electrodes in active electronic components, enabling the fabrication of various stretchable and self-healing sensors and displays. Beside multiple hydrogen bonding, multiple supramolecular metal-ligand interactions are also demonstrated to fabricate self-healing elastomer. Bao and coworkers reported a supramolecular elastomer composed of coordination complexes crosslinked PDMS polymer chains [151]. The crosslinking sites were formed based on 2,6-pyridinedicarboxamide ligands coordinating to Fe(III) centers, which contained three different interactions including a strong pyridyl-iron bond, and two weaker carboxamido-iron ones coordinated with nitrogen and oxygen atoms of the carboxamide groups. The stronger coordinate interaction with pyridyl ring enabled reversible unfolding and refolding of the chains, while the weaker ones endowed the material with desirable self-healing performance. The resultant elastomer showed high stretchability (4500 ± 20% strain) and desirable self-healing properties at low temperature (−20 °C) without influenced by surface ageing or moisture.

Another promising approach is to introduce multiple dynamic crosslinking network with different types, which would combine respective advantage of various chemical bonds [152]. For the purpose of improving the poor material mechanical property of hydrogen bonding, Jia and coworkers fabricated an elastic autonomous self-healing capacitive sensor via introducing a dynamic dual crosslinked chemical system (Figure 5b) [153]. The dynamic metal-coordinated bonds (β-diketone-europium interaction) and hydrogen bonds were specially designed in polymeric PU matrix. The resultant materials formed a microphase-separated structure and showed a high tensile strength of 1.8 MPa and high fracture strain of about 900%. Despite desirable mechanical properties, this material required more healing time (48 h) to realize a self-healing efficiency of 98% at 25 °C after mechanical break. Moreover, Xu and coworkers synthesized a self-healable conductive polymer composite with high stretchability (≈500%) and electrical conductivity (0.12 S cm^−1^) [154]. This composite comprised of three components including PANI, PAA and phytic acid, which played roles of conductive elements, soft chains and conductive dopants, respectively. These components would interact with each other to form dynamic hydrogen bonding and electrostatic interactions. On rupture, both mechanical and electrical properties of this material could achieve a self-healing efficiency of >99% within 24 h at room temperature due to the collective contributions from this dual supramolecular dynamic interaction. This self-healing conductive polymer composites with high sensitivity to strain and pressure demonstrated great potentials in e-skin application.

Moreover, the afore-mentioned strategies for multiple dynamic crosslinking network construction are not only suitable for solid polymer material, but also works in polymer gel system [155,156]. For example, Wu and coworkers fabricated a biomimetic iontronics combining a wide spectrum of mechanical properties and multiple sensory capabilities (Figure 5c) [81]. The supramolecular polyelectrolyte hydrogels were utilized as the fundamental material for biomimetic skin. The multiple dynamic crosslinks in supramolecular hydrogel, including hydrogen bonds and ionic associations and hydrophobic interactions, made it possible to quickly heal multiple detecting capabilities toward temperature, strain and stress under ambient environment after damages. Although this material exhibited excellent skin-like functionalities, the mechanical property of it was relatively poor. Moreover, Sun and coworkers demonstrated a mechanically robust, elastic and healable ionogels for preparing highly sensitive and reliable ionic skins (Figure 5d) [157]. The poly(urea-urethane) dynamically crosslinked by reversible urea bonds and hydrogen bonds was chosen as the polymer matrix. After impregnating ionic liquids into this polymer, an ultra-durable ionic skin was obtained, which was highly sensitive to a wide range of strains (0.1–300%) and pressures (0.1–20 kPa). Such a dual dynamic crosslinking network endowed the as-prepared ionogels with good elasticity, high mechanical strength and Young’s modulus similar to that of natural skin. However, external heating (65 °C) was required to completely heal fractured I-skin. Besides ionogels materials, hydrogel-based conductive materials could also be employed to fabricate self-healable electronic sensors. However, owing to the freezing of water under subzero-temperature and the evaporation of hydrogel, it is difficult to design self-recoverable hydrogel-based e-skin which can heal at low temperature and possess minimum water-loss at ambient temperature. Inspired by the fiber-reinforced microstructure and mechano-transduction systems of human muscles, Dong and coworkers proposed a self-healable, long-lasting thermal tolerant and dual-sensory hydrogel-based stain and temperature sensor consisting of multiple supramolecular bonds crosslinked conductive PANI nanofibers/PAA composites [158]. The metal ions (Fe^3+^), strong hydrogen bonding and electrostatic interactions presenting in this material system synergistically contributed to desirable mechanical strain (~991%), area expansion (~1500%) and satisfactory self-healing properties (healing efficiency: 90.8%). A glycerol/water binary solvent system was specially introduced to improve subzero-temperature self-healing performance (−26 °C), high water-retaining property and durable adhesion feature. Moreover, Yu and coworkers reported a flexible conductive organohydrogel-based strain sensor comprising of MXenes blended polyacrylamide and polyvinyl alcohol (PVA) polymer networks, which featured excellent anti-freezing, long-lasting moisture, self-healing capabilities and superior mechanical properties, as well as satisfying sensing performance (Figure 5e) [159]. By means of relacing a portion of water solvent with ethylene glycol (EG), the resultant hydrogel showed a strong anti-freezing capability at extreme temperature (−40 °C) and long-lasting moisture retention (8 days). The hydrogel also exhibited excellent self-healing capability, which was enabled by the dynamic crosslinking between the hydroxyl group of PVA and tetrahydroxyl borate ions, and the supramolecular hydrogen interaction among EG, PVA and MXene. In addition, the hydrogel-based strain sensors were able to detect human biologic activities with a relatively broad strain range (up to 350% strain) and a high gauge factor of 44.85 in real time, even under extremely low temperatures.

### 3.3. Covalent-Noncovalent Interpenetrating Network

**Figure 6:**
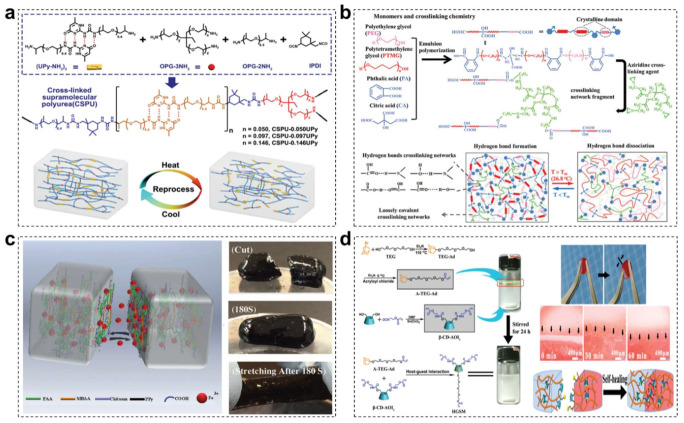
(**a**) The synthesis of supramolecular polyureas and chemical structures of the designed noncovalently bonded diamine monomers, covalent diamine/triamine monomers and diisocyanate monomers. Schematic diagram of the recycling process of the resultant polyureas based on the covalent-noncovalent crosslinking strategy [162]. Copyright 2020, Wiley-VCH. (**b**) The monomers, prepolymers and crosslinking agents used in the synthesis of copolyester, and a schematic network fragment of the copolyester. Schematics of the network structure before and after crystallization-melting transition. Reproduced with permission [163]. Copyright 2019, Wiley-VCH. (**c**) Schematic healing mechanism of the resultant hydrogel physical and chemical crosslinking networks. Digital pictures during cut, healing for 180 s and stretching after healing. Reproduced with permission [167]. Copyright 2017, Wiley-VCH. (**d**) Schematic synthesis procedures of self-healing hydrogels with dynamic host-guest noncovalent crosslinking network and covalently linked network design. Right pictures are corresponding self-healing process photos. Reprinted with permission from ref. [168]. Copyright 2019, Royal Society of Chemistry.

Compared with noncovalent bonds, covalent ones normally possess higher bond strength but exhibit poor reversibility, which means that they are difficult to reconstruct and sustain original mechanical properties once experiencing breaking. To address this irrecoverable issue of covalent bonds, dynamic noncovalent bonding is introduced to form covalent-noncovalent interpenetrating network, which endows the material system with desirable self-healing capacity and satisfying mechanical robustness simultaneously [160,161]. For example, Zhang and coworkers demonstrated a tough and multi-recyclable cross-linked supramolecular polyureas via introducing noncovalent quadruple hydrogen bonds-linked diamine monomers and covalent diamine/triamine monomers (Figure 6a) [162]. With the novel covalent-noncovalent crosslinking network design, the synthetic supramolecular polyureas exhibited remarkable solvent resistance and excellent mechanical properties (with a tensile strength above 30 MPa and a superior toughness of MJ m^−3^). Impressively, the resultant material would recover more than 95% of their original properties even after five complete damage/recycling processes, benefitting from the reversible nature of quadruple hydrogen bonds. Despite satisfying recovered mechanical performance, the self-healing effect of these covalent-noncovalent crosslinking materials is inevitably satisfied and more strict healing condition is required to enhance the chain mobility, owing to the irreversible characteristic of covalent crosslinking network. To handle this problem, our group recently fabricated a novel biopolyester with a dual covalent-noncovalent interpenetrating crosslinking network for hygroscopic robot application (Figure 6b) [163]. Firstly, a macromolecular prepolymer with low melting point was elaborately synthesized through the condensation copolymerization reaction of poly(ethylene glycol) and poly(tetramethylene glycol) precursor and then cured by a small percentage of the tri-arm crosslinking agent to form a loosely covalent crosslinking network. At the same time, the abundant carboxyl groups and hydroxyl groups were likely to react with each other to form dense multiple supramolecular hydrogen bonding networks. Thanks to the special covalent-noncovalent dual networks with diverse crosslinking density, the resultant biopolyester presented robust mechanical properties, excellent self-healing ability (with a healing efficiency of nearly 100%), straightforward manufacturability at low ambient temperature (≤35 °C), as well as fast and stable hygroscopic actuation.

In conductive hydrogel material system, the covalent-noncovalent crosslinking strategy is also commonly used to endow them with high-performance self-healing capacity [164,165,166]. For example, Xing and coworkers developed a mechanically and electrically self-healing hydrogel based on dual physical and chemical crosslinking networks (Figure 6c) [167]. The covalent cross-linking played an important role in supporting the mechanical structure of the hydrogel, while the supramolecular ionic interactions between carboxylic groups of PAA and ferric ions were used to endow the hydrogel with autonomous intrinsic self-healing ability. The self-healable hydrogel with nanostructured conductive PPY networks design exhibited bulk conductivity, ultra-stretchability (1500%), mechanical and electrical self-healing properties (100% mechanical recovery in 2 min), as well as pressure sensitivity, which demonstrated practical applications in wearable strain and pressure sensing devices. In addition, Mao and coworkers adopted the same covalent-noncovalent crosslinking strategy to fabricate 3D-printable self-healing and mechanically reinforced hydrogels via introducing host-guest noncovalent interactions into covalently linked networks (Figure 6d) [168]. In this hydrogel material, the covalent crosslinking could maintain its overall shape, whereas the weak dynamic non-covalent host-guest interactions were able to reinforce its mechanical properties and enabled it to rapidly self-heal once fracturing. In addition to self-healing property, the reversible non-covalent interactions also played roles of dissipating energy to prevent fracture propagation. This robust, fatigue resistant, 3D-printable, biocompatible and self-healable hydrogels shows great potentials in biomedical applications.

### 3.4. Interfacial Supramolecular Crosslinking Networks

As discussed above, electric conductive composites based on conductive fillers mixed polymer matrix with sensitive micro/nano-structure design are prospective materials for wearable electronic devices. The generated interfaces between fillers and polymer chains are the weakest sites. These fragile interfaces are vulnerable to slippage and damage under repeated and complex stress condition, which will cause the unstable signal even the whole device break down. For the purpose of improving the stability and reliability of the whole sensors, it is important to build robust but reversible interfaces in conductive composites-based sensing materials. However, the previous reported covalent-bonded interface for electronic sensor application lacks dynamic characteristic, which is unable to restore original performance after experiencing unexpected damage. To solve this problem, interfacial supramolecular crosslinking networks are creatively introduced into conductive polymer composites, making it possible not only to improve mechanical properties and performance stability, but also to endow wearable e-skin with outstanding self-healing capacities [169,170,171,172].

**Figure 7:**
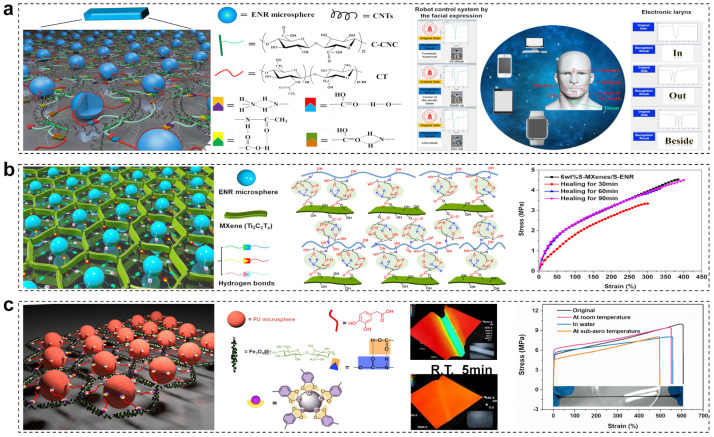
(**a**) Proposed supramolecular multiple hydrogen bonding network combined with nanostructured CNT conductive network design in ENR latex microspheres. Robot control system by the facial expression and electronic larynx based on the self-healing strain sensors. Reproduced with permission [173]. Copyright 2017, Wiley-VCH. (**b**) Interfacial supramolecular hydrogen bonds combined with the 3D segregated conductive networks design in NMSE. Corresponding chemical structure of interfacial hydrogen bonds between S-MXene nanosheets and S-ENR chains. Tensile curves of resultant self-healing elastomer at different self-healing times. Reprinted with permission from ref. [174]. Copyright 2020 American Chemical Society. (**c**) Schematic illustration for hierarchical structural design and interfacial supramolecular crosslinking in PU matrix. The super depth of field microscope images of samples before and after healing process. Stress-strain curves of samples healed at room temperature and under different harsh conditions. Reproduced with permission [176]. Copyright 2019, Wiley-VCH.

Inspired by the multiple hydrogen bonding connection of the deoxyribonucleic acid (DNA) double helix to attain ultrafast self-healing ability and mechanical robustness, our group demonstrated a highly sensitive and self-healable electronic sensor enabled by interfacial supramolecular hydrogen bonding and nanostructured conductive network design (Figure 7a) [173]. Bio-derived carboxyl cellulose nanocrystals (C-CNC) were specially utilized to construct multiple interfacial hydrogen interactions with chitosan-decorated epoxy natural rubber (CT-ENR) latex. The abundant carboxylic and hydroxy groups in C-CNC molecular chains could interact with the rich amino, acetamide, and hydroxy groups in CT-ENR to form multiple interfacial hydrogen crosslinking networks. In the meanwhile, amphipathic C-CNC could assist the assembly of CNTs to construct a 3D nanostructured conductive network during the latex assembly process. Taking advantages of this conductive supramolecular elastomer, a nanostructured electronic sensor was specially fabricated via electrostatic layer-by-layer assembling process, which featured high sensitivity, low detection limit, extremely fast healing time (15 s) and repeated self-healing ability with high healing efficiency (93% after the third healing process) under ambient temperature. Then, a facial expression control system and electronic larynx were developed via integrating a human-machine interaction system with this high-performance sensor, showing great potentials in intelligently control robots and help the mute to speak again. Based on similar interfacial dynamic crosslinking mechanism, we further reported a self-healable nanostructured Ti_3_C_2_ MXenes/rubber-based supramolecular elastomer (NMSE) for intelligent sensing materials (Figure 7b) [174]. Specifically, serine, an amino acid containing amino, carboxyl and hydroxyl groups, was specially used to modify both MXene nanoflakes and ENR latex via esterification and ring-opening reaction. The unreacted serine residues were able to associated with each other to form dynamic hydrogen bonding interfaces between MXene fillers and ENR matrix. Thanks to the dynamic nature of hydrogen bonds and nanostructured conductive network design, the resultant NMSE-based sensors exhibited high gauge factor (107.43), low train detection limit (0.1%), fast response time (50 ms), as well as desirable recovered tensile strength (4.52 MPa) and excellent self-healing performance (~100%) at room temperature.

Moreover, interfacial supramolecular metal-ligand coordination can also be employed to endow composite materials with desirable self-healing performance. For example, our group designed a self-healing electronic sensor with tunable positive/negative piezoresistivity based on hierarchically structured conductive network and supramolecular metal-ligand coordination crosslinking network design [175]. ZnCl-decorated ENR and histidine (His)-modified CNC were utilized to construct dynamic metal-ligand coordination crosslinked interfaces via a reversible coordination reaction between His and Zn^2+^. At the same time, amphiphilic CNC would act as a bio-template to disperse neat CNTs and mixed with ENR-Zn latex to obtain conductive nanostructured supramolecular elastomers. They exhibited autonomous, fast (2 min) and repeatable self-healing ability with high healing efficiency (88.6% after the third healing process). The healed samples still possess stretchability, high sensitivity, and accurate detection capability even after bending over 10,000 cycles, making it promising for next-generation wearable electronics. Moreover, the strategy of interfacial supramolecular crosslinking network is also suitable for fabricating other self-healable functional composite materials. Recently, we fabricated a hierarchically structured self-healing composite for ultra-fast light- and magnetic-response actuator application (Figure 7c) [176]. At first procedure, well-dispersed Fe_3_O_4_@CNC nanohybrids were prepared through in situ growing Fe_3_O_4_ NPs on CNC nanorods, which was efficient magnetic and photothermal conversion component. Then, 3,4-dihydroxyphenylacetic acid (DOPAC) was utilized to construct interfacial supramolecular crosslinking in PU matrix via metal-ligand coordination between Fe_3_O_4_ NPs and catechol groups of DOPAC. The resultant elastomer showed excellent self-healing performance after damage, benefitting from this interfacial supramolecular crosslinking structure. Furthermore, the 3D interconnected network design of Fe_3_O_4_@CNC design in PU substrate promoted the realization of ultrahigh photothermal conversion efficiency, rapid and reversible infrared light/magnetic field-responsive actuating performance.

**Figure 8:**
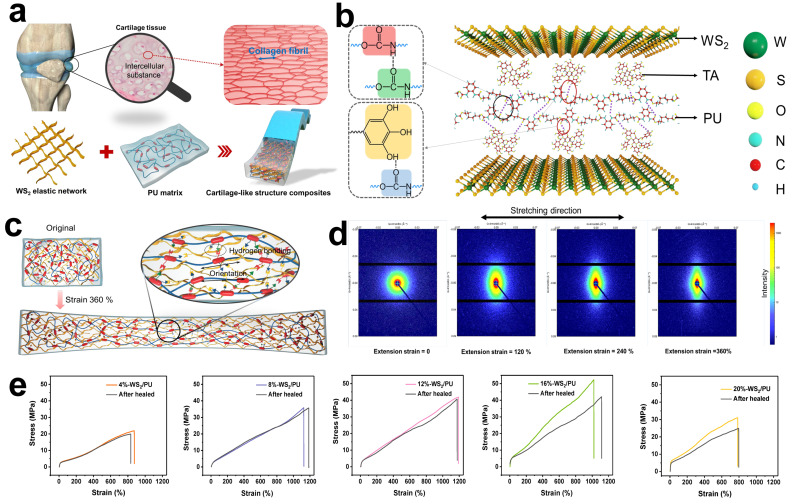
(**a**) Schematic illustrations of a cartilage structure, intercellular substance of cartilage tissue and the nanostructure of composite consisting of hydrogen-bonded interwoven network of 2D WS_2_ and PU matrix. (**b**) Schematics of the dynamic noncovalent bonding interaction between PU and interwoven network of 2D WS_2_. (**c**) Schematic illustrations of the nanostructure of the original sample and stretching sample. (**d**) 2D SAXS images of the 16 wt% TA-WS_2_/PU with different tensile strain during uniaxial stretching process. (**e**) Mechanical self-healing performance of PU composites filled with different contents of TA-WS_2_. Reproduced with permission [177]. Copyright 2021 Springer Nature.

Very recently, we creatively demonstrated an ultra-robust self-healing elastomer, which overturns the previous perception that high mechanical robustness and healing ability are mutually exclusive in noncovalent bonding self-healing materials [177]. In nature, human’s cartilage tissue is an extraordinary structural material, comprising of collagen cells and intercellular substance (Figure 8a). The interwoven collagen fibers and abundant hydrogen bonding network between these two components synergistically contribute to high mechanical strength and certain self-healing ability. Drawn inspiration from this hierarchical structure presenting in cartilage tissue, we designed a high-performance self-healing elastomer by means of introducing high-density interfacial noncovalent bonds among the dendritic tannic acid-modified tungsten disulfide (TA-WS_2_) nanosheets and PU matrix (Figure 8b). Owing to the presence of strong interfacial supramolecular interactions, the as-prepared nanocomposite with interwoven network not only showed ultra-high mechanical performance (a tensile strength of 52.3 MPa, high toughness of 282.7 MJ m^−3^, and stretchability of 1020.8%), but also surprisedly possessed an excellent self-healing properties (a healing efficiency of 80–100%), surpassing nearly all the reported self-healing materials (Figure 8e). Interestingly, we found that the plentiful interfacial hydrogen bonds facilitated the orientation of PU chain and cause strain-induced self-reinforcement during stretching process, as illustrated by the 2D SAXS images and schematic diagrams (Figure 8a,b). Therefore, the physical crosslinking network and self-reinforcement of our microscale/nanoscale-assembled cartilage-like nanocomposites dramatically promoted the improvement of mechanical properties. We believe that this work will open a promising route to explore ultra-robust self-healing materials for various functional devices, and the design concept presented here provides insightful guidance to fabricate high-performance self-healing composites.

## 4. Summary and Outlook

Nature is the everlasting inspiration source for the exploration of intelligent devices with new structures, functions, and materials. In this review, we highlight recent progress in multiple-stimuli-responsive e-skin with self-healing properties, focusing on the material design and fabricating strategy for specific bionic functionalities including multiple stimuli sensing and self-healing capacities. E-skins equipped with these fascinating bionic functionalities makes them attractive and competitive for next-generation skin-like smart devices. Multiple-stimuli-responsive electronic sensors could simultaneously detect physical force, physical chemistry parameters and multiple biochemical signals and thereby provide abundant environmental information or comprehensive physiological health information for disease diagnosis, healthcare, and motion monitoring applications. It is especially crucial in this fast era as the risk of illness and injury are gradually increased. The perception of multiple stimuli is also accompanied with signal interference problem and relative decoupling technology or material designed concept have been included in this section. Moreover, developing e-skins with self-healing capacity is also of great significance to their performance stability and service time. One can envision exciting possibilities that self-healable e-skin could completely recovered even after suffering repeated mechanical damage and wouldn’t occur performance failure similar to an immortalized material. This research direction has attracted increasing attentions and great progress has been achieved in recent decades.

However, despite brilliant achievement, there are still some challenges remained to be solved for next generation e-skin. In the following content, we briefly discuss these challenges for each biomimetic functionality. (1) For multiple stimuli-sensitive e-skins, most efforts have been made to explore novel stimuli-responsive behavior and improve their sensitivity or sensing range. Nevertheless, there are still huge obstacles for the integration of multimodal sensing components in one e-skin devices. Normally, electric sensors capable of detecting single temperature, humidity, force or biochemical stimuli rely on completely different response mechanism and correspond to diverse material structure design and fabrication process. Thereby, it puts forward more stringent requirements for the integration of these individual elements, such as circuit layout, compositional compatibility, and large-area fabrication. Furthermore, the selectivity and signal interference problem should obtain sufficient attentions for multimodal electronic sensors. As multiple information is collected from the same e-skin, effective but facile signal decoupling technologies and accurate discrimination of different stimulus inputs are highly desirable for multimodal e-skins, especially for multiple biochemical sensors that are highly sensitive and are likely to interference by the other stimuli. (2) Self-healable e-skins with rapid and autonomous self-healing capacity at ambient temperature after mechanical damage are of great importance to sustain their performance stability and reduce maintenance cost. Intrinsically self-healing materials for e-skin materials often rely on the mobility of polymer chain and reversible characteristic of dynamic crosslinking network. However, these self-healable polymer matrix with low glass-transition temperature suffer from high hysteresis and slow response/recover time problems which restrict the realization of real-time monitoring in e-skin. Moreover, in most conductive self-healing system, it is still challenging to solve the contradiction between mechanical performance and healing efficiency. The self-healing material with high mechanical strength often exhibits limited healing efficiency and requires strict recovering condition. As a result, the application of self-healable materials to flexible electronic is still as proof-of-concept state. In addition, the development of self-healable materials that can heal under extreme conditions, such as underwater, acid, alkaline, electric, and aerospace environment, are still hard to achieve. If these challenges would be successfully addressed, we envisage that it will greatly broaden the widespread application of self-healable multifunctional e-skin and promote the booming development of IOTs.

## Data Availability

The review used information from published studies, which are referenced accordingly.
